# Everyday Problem Solving and Instrumental Activities of Daily Living: Support for Domain Specificity

**DOI:** 10.3390/bs3010170

**Published:** 2013-03-07

**Authors:** Kristopher J. Kimbler

**Affiliations:** Department of Social and Behavioral Sciences, Florida Gulf Coast University, 1051 FGCU Blvd. S. Fort Myers, FL 33965, USA; E-Mail: kkimbler@fgcu.edu; Tel.: +1-239-590-7425; Fax: +1-239-590-7445

**Keywords:** cognitive aging, everyday problem solving, instrumental activities of daily living

## Abstract

Research suggests that performance on cognitive tasks resembling daily challenges (*i.e.*, everyday problem-solving tasks) may be a better indicator of functional ability in old age compared to traditional measures of cognitive ability. Findings demonstrating this link, however, have yielded mixed results. The current study examined performance on the Everyday Problems Test (EPT) and self-reported ability to perform Instrumental Activities of Daily Living (IADLs) in a sample of adults over age 50. The EPT measures cognitive performance on tasks with domains consistent with IADLs (telephone use, shopping, meal preparation, housekeeping, transportation, health and finances). Although overall EPT scores and self-reported IADLs were significantly related (*r_s_* = 0.20; *p* < 0.05), additional analyses revealed that domain-specific EPT performance related to IADL reports within the same domain for shopping, meal preparation, housekeeping, and financial management after accounting for other variables such as age, sex, and measures of cognitive ability including total EPT score. These findings suggest that domain-specific performance on cognitive everyday problem-solving tasks may add to the predictability of specific IADLs.

## 1. Introduction

The current study attempts to illustrate a potential gain of utilizing a domain specific approach to the study of cognitive ability related to everyday problem solving and functional ability among middle-aged and older adults. While cognition commonly refers to mental processes related to acquiring, storing and using information, the term cognitive ability usually describes individuals’ performance on cognitively demanding tasks. These cognitive processes are not directly observed but are typically inferred based on behavioral outcomes such as performance of cognitive tasks. Cognitive tasks refer to performance-based measures that are primarily intended to demonstrate the knowledge and thought processes required to successfully complete the task [1]. Although there are a wide variety of measurement options for assessing specific domains of cognitive ability, there tends to be some consistency regarding the types of task demands across different measures within specific domains of cognitive ability. For example, some domains, such as fluid and crystallized abilities, have been examined utilizing different established measurement options and research suggests that there is considerable overlap in variance and predictive potential related to these measures commonly used as indicators of these cognitive abilities [2]. These cognitive ability concepts allow researchers to group tasks and measures that require similar mental processes for successful task completion. To describe only the phenomenon being observed, such as performance on a specific task, would ignore the extent that previous research has supported the claim that the task requires specific cognitive abilities (e.g., knowledge, reasoning, memory, *etc.*) that extend beyond the scope of a specific measure / observable behavior. Some cognitive domains, however, are less consistently defined. Everyday problem-solving tasks represent a type of cognitive task that is intended to assess cognitive ability relevant to day-to-day challenges. The current study attempts to demonstrate that these tasks are more complex and multifaceted than many other types of cognitive tasks and that there is potential benefit in adopting a more domain specific approach when analyzing performance on these types of tasks and linking this performance to other outcomes.

### 1.1. Cognitive Ability and Aging

A great deal of research examining cognition in old age has focused on different types of cognitive abilities that display a variety of developmental trajectories [3,4]. Although age related declines are exhibited in many aspects of cognitive ability, many older adults are still able to function independently without the need for daily assistance from others [5,6]. These findings suggest that cognitive decline may not affect functional ability until the declines become more pronounced. Previous research has attempted to explain the extent that different types of cognitive ability and decline relate to older adults’ functional abilities [7]. The current study examines indicators of fluid abilities (inductive reasoning), crystallized abilities (verbal ability), and everyday problem-solving performance as indicators of middle-aged and older adults’ ability to perform instrumental activities of daily living.

Research examining cognitive ability in adulthood has provided strong evidence suggesting that cognitive ability should not be represented by a single construct such as general intelligence (*g*). Instead, specific abilities such as crystallized and fluid abilities, memory, processing speed, and executive function provide much more insight into developmental changes in cognitive ability [8]. The current study focuses on crystallized and fluid abilities. The distinction between fluid and crystallized abilities largely relates to whether or not a cognitive task involves the utilization of experience-based knowledge (crystallized abilities) or the utilization of reasoning and problem-solving skills to solve novel problems (fluid abilities), not dependent on prior experiences [9,10]. 

The distinction between these types of abilities is evident when examining the different developmental trajectories across adulthood that are consistently found for fluid and crystallized abilities. Fluid abilities typically exhibit an earlier, more distinct decline. Crystallized abilities, however, demonstrate greater stability across adulthood and can even increase into old age [2,4]. It is important to note, however, that although these changes in fluid abilities are considered to be normative shifts in cognitive ability, research has suggested that efforts made through interventions and training can be successful at slowing or reversing this type of decline [11]. 

### 1.2. Everyday Problem Solving

Another approach to studying cognitive ability that has received a great deal of recent attention involves everyday problem solving. Everyday problem-solving tasks are an attempt to assess individuals’ ability to solve cognitively challenging questions that they are likely to encounter throughout their daily life [12]. This is believed to be an ecologically valid approach to studying cognitive ability in old age, as it examines the individuals’ ability to apply their knowledge and problem-solving skills to demonstrate the extent that they can reach successful solutions to the types of age-appropriate problems they may face throughout their life. Everyday problem-solving tasks also have varying domains and formats. Some of these tasks involve ill-defined questions with multiple possible solutions while other tasks are well defined with only one correct solution [7]. In addition, these task domains can include problems ranging from social relationship dilemmas to instrumental activities such as managing finances.

This range of domains and formats, combined with a lack of longitudinal data, makes it difficult to establish definitive conclusions regarding age related changes in everyday problem-solving performance. Still, many studies have examined age differences and aging issues using cross-sectional designs in everyday problem solving [12,13,14]. In an attempt to compile many of these findings, Thorton and Dumke [15] conducted a meta-analysis examining everyday problem-solving performance and age. This meta-analysis suggested that the performance of middle-aged and younger adults exceeded the performance of older adults on everyday problem-solving tasks. Other studies have also found an older adult deficit but suggested that performance in everyday problem-solving tasks may peak during middle adulthood [16,17]. 

### 1.3. Cognitive Ability and Everyday Problem Solving

Everyday problem-solving performance does appear to be related to more standard measures of cognitive ability. Specifically, research has found significant correlations between performance on well-defined everyday problem-solving tasks and performance on tasks of fluid abilities, crystallized abilities, memory, and perceptual speed [7,18]. It is therefore plausible that individuals utilize a variety of cognitive skills when completing everyday problem-solving tasks. Relating specifically to crystallized and fluid abilities, the familiarity of the domains used in everyday problem solving could suggest that experience-based knowledge (crystallized abilities) could assist in reaching a successful solution. These questions are also likely to have components or specifications that participants have not directly encountered. This novel component would benefit from fluid abilities.

### 1.4. Instrumental Activities of Daily Living (IADLs) and Cognitive Ability

Instrumental activities of daily living (IADLs) have received a great deal of research attention. These activities involve skills that are required to maintain complete independence. An inability to complete IADLs suggests that an individual needs some assistance from either informal social support or from formal support services. IADLs are reliable indicators of functional ability and the less-severe forms of disability that are typically found in community-dwelling older adults [19]. These tasks (such as telephone use, shopping, meal preparation, housekeeping, laundry, transportation, medication responsibility and finances) can require both physical and cognitive demands for successful completion [20]. 

Research examining the link between cognitive ability and functional ability (*i.e.,* IADL performance) has yielded mixed results. Allaire and Marsiske’s [7] research suggested that performance on traditional measures of cognitive ability (including tasks related to fluid and crystallized ability) did relate to self-reported measures of functional ability, including Lawton and Brody’s measure of IADLs [21]. Performance on cognitive everyday problem-solving tasks, however, mediated this relationship and also accounted for additional variance in self-reported functional ability. Other research, however, has suggested that common measures of functional ability, such as Lawton and Brody’s [21] self-report IADL measure, may lack sensitivity in detecting slight declines in functional ability related to mild cognitive impairment [22,23]. Still other studies indicate that several components of cognitive ability, such as executive function, mental status, verbal ability, and cognitive impairment status relate to IADL performance [23,24,25]. Particularly relevant to the current study, Willis and colleagues [26] found that cognitive training in inductive reasoning (a fluid ability) was related to a significant improvements in self-reported IADL performance while training in speed of processing and memory did not result in a significant benefit in IADL performance. 

Related to everyday problem solving and functional ability, one study suggests an unclear link. Schmitter-Edgecombe, Parsey, and Cook [27] found that everyday problem-solving performance on the Everyday Problems Test and self-reported functional ability (IADLs) were both significantly related to observational measures of IADLs. Everyday problem-solving performance and self-reported functional ability, however, were not significantly related. It was suggested that this lack of a correlation between the self-report and the everyday problem-solving measure could result from these measures explaining different components of functional ability. Specifically, the everyday problem-solving task relates to cognitive dimensions of functional ability while the self-report measures could relate to physical, cognitive, and health related components of functional ability. 

This lack of consistency in previous research examining the relation between everyday problem-solving performance and functional IADL ability indicates that there is a need for more research to better clarify the extent of this relationship. It also suggests that many factors such as sample characteristics and cognitive impairment may be important in clarifying the extent that everyday problem-solving performance relates to functional ability. 

Most previous research has focused primarily on overall scores of self-reported functional ability and everyday problem solving. A study conducted by Allaire and colleagues [23] utilized a more domain specific approach to examining the role of mild cognitive impairment and IADLs, as indicated by self-report measures and everyday problem-solving performance. This study focused on the domains of medication use, finances, and nutrition / food preparation. They found that participants with mild cognitive impairment differed from other participants on all three subscales of the memory-based everyday problem-solving task. This study also found differences when comparing those with and without cognitive impairment related to self-report measures of IADL ability in the domains of finances and meal preparation but no significant differences in domains related to medication and shopping. In addition, after accounting for demographic and cognitive variables, the memory-based everyday problem-solving task still significantly predicted cognitive impairment in the medication and finance domains, but not the food domain. The current study attempts to extend this domain specific approach by examining the relation between domain-specific, reasoning-based everyday problem-solving performance and domain specific functional ability as indicated by self-reports of IADL performance across a variety of domains. 

### 1.5. Specific Aims and Hypotheses

The first objective of the current study was to examine the relation between overall self-reported functional ability (IADLs) and overall everyday problem-solving performance. As previously described, the aforementioned research has yielded mixed results, demonstrating inconsistent correlations between these types of measures. A specific study utilizing the Everyday Problems Test and a sample of community-dwelling adults aged 50 and older found no significant relation to self-reported functional ability [27]. It was therefore hypothesized that the two measures would not be significantly related.

The second objective was to assess the extent that a domain-specific approach would strengthen the relation between everyday problem-solving performance and self-reported functional ability (IADLs). It was hypothesized that this domain-specific approach would increase the extent that these two variables are related. Previous research has demonstrated that different IADL domains do require different physical and cognitive skills [20]. It was therefore expected that individuals reporting high abilities within a specific IADL domain would perform better on everyday problem-solving tasks within the same domain due to an increased familiarity with the demands and strategies required to successfully complete the task. It was also hypothesized that this domain-specific approach would explain variance in self-reported functional ability (IADLs) after accounting for age, sex, fluid ability (indicated by an inductive reasoning task), crystallized ability (indicated by a verbal ability task), and overall everyday problem-solving performance. 

## 2. Methods

### 2.1. Participants

Participants (*N* = 102) were recruited using advertisements, participant referrals, and by contacting different organizations (e.g., civic organizations and churches) in an eastern state within the United States. Participants recruited for participation in this study included middle-aged and older adults age 50 and older. This age group was targeted due to previous research suggesting that performance on cognitive everyday problem-solving tasks may begin declining after age 50 [12] and is more pronounced by age 70 [15]. In addition, previous studies have utilized samples aged 50 and older when examining everyday problem solving, cognitive ability, and self-reported IADL ability [27]. To be included in the study, all participants had to report living independently within the community. Individuals living in assisted living or nursing home environments were not included in the study.

Participants ranged in age from 50 to 92 (*M* = 65.56, *SD* = 10.94). Middle-aged participants (aged 50–59) comprised 37.25% of the sample (*n* = 38; 60.5% male, 39.5% female), young-old participants (aged 60–74) comprised 39.22% of the sample (*n* = 40; 45% male, 55% female), and old-old (aged 75 and older; 45% male, 55% female) comprised 23.53% of the sample (*n* = 24). Examination of the educational attainment of participants revealed that 44.10% of participants reported receiving some form of education or training beyond high school, 33.33% reported earning only a high school degree, and 22.55% reported not graduating high school. The sample did not demonstrate a large amount of variability related to race and ethnicity as 95% of participants reported being White / Caucasian, 3% reported being Black / African American, and 2% reported other ethnic group membership. Participants also selected an income range that best represented their household income. The median income range was $30,000–$50,000.

### 2.2. Measures

#### 2.2.1. Inductive Reasoning

The Letter Series Test [28] was administered to measure inductive reasoning ability. This task is also considered an indicator of a fluid cognitive ability and is negatively correlated with age [4]. During this task, participants were provided 30 items that contain a series of letters. Participants attempted to identify the letter that would appear next in the pattern out of five possible choices. Participants completed as many items as they could within six minutes. The score was calculated by summing the number of correct responses. 

#### 2.2.2. Verbal Ability

The Verbal Meaning Test [29] was administered to measure participants’ verbal ability. This task is considered an indicator of crystallized cognitive ability and performance is typically positively correlated with age [30]. During this task, participants are provided with a stimulus word and are asked to select the word out of five choices with the same meaning as the stimulus word. Participants were provided three minutes to complete as many of the 30 items as possible. The score was calculated by summing the number of correct responses. 

#### 2.2.3. Everyday Problem Solving

The Everyday Problems Test (EPT) [31] was administered to indicate participants’ ability to solve problems related to the domains of telephone use, shopping, meal preparation, housekeeping, transportation, health / medication use, and finances. These domains mirror the IADL domains but this measure assesses participants’ ability to solve cognitively challenging everyday tasks relevant to these domains, not their actual functional ability to perform these tasks in a natural setting. The current study combined two short forms of the EPT, resulting in a total of 28 items [32]. During the EPT, participants were provided two stimuli related to each of the seven domains. They were then asked to solve two problems based on each stimulus. Although responses were open-ended, this is a well-defined task with only one correct response to each question. The information provided in the stimuli includes all content needed to generate the correct response to each question. The score on the EPT was calculated by summing the number of correct responses out of the 28 items. In addition, domain-specific scores were calculated by summing the number of correct responses within each domain. There were four items and two stimuli representing each of the seven domains. 

#### 2.2.4. Instrumental Activities of Daily Living (IADLs)

Participants completed the Lawton and Brody IADL scale [21] as an indicator of self-reported functional ability. This questionnaire requires participants to provide self-reported ratings on 3 to 5-point Likert scales indicating their ability to perform IADL tasks. The IADL domains included in this questionnaire consist of telephone use, shopping, meal preparation, housekeeping, laundry, transportation, medication responsibility, and finances. Similar to the scoring technique used by Ng, Niti, Chiam, and Kua [20], participants’ responses for each domain were scored 1, 2, or 3. A score of 1 indicates that the participant is completely unable to perform the task. A score of 2 indicates that the participant can perform the task but requires some assistance. A score of 3 indicates that the participant can perform the task independently, without any assistance. The total score was calculated by summing the scores of all eight items. Although this measure relies on self-report, it is one of the most common measures used to assess functional ability in research [22] and is used in applied settings [33]. Also, previous studies have revealed that it is a useful measure of functional ability and can predict observation-based measures of IADL performance [27].

### 2.3. Procedure

Data for the current study was collected as part of a larger study examining cognitive ability [34]. Participants were scheduled to meet with the researcher individually. After signing the informed consent and verifying that they were at least 50 years old, participants completed the Verbal Meanings Test [29] and the Letter Series Test [28]. Participants then completed a 28-item version of the Everyday Problems Test [31]. During the completion of each cognitive test, the researcher left the room so the participant could complete the test alone and without distraction. After all tests of cognitive abilities were completed, participants were given a questionnaire and were asked to provide information including age, income, education, race / ethnicity, and functional ability.

## 3. Results

### 3.1. Descriptive Data

Table 1 reveals mean total and domain specific scores for reported IADL ability and EPT scores as well as verbal ability, inductive reasoning, and education for middle-age, young-old, and old-old participants. As indicated by the table, the sample is a high-functioning sample, as evidenced by a ceiling effect related to self-report IADLs. The other variables reported in the table were normally distributed. The overall mean IADL score was high (*M* = 21.72; *SD* = 2.72) out of a maximum possible score of 24. As indicated by Table 1, mean IADL scores were high for all 3 age groups (ranging from 21.13 to 22.23). Remaining analyses will account for this skewed IADL data.

**Table 1:** Mean scores for middle-aged, young-old, and old-old participants in IADL reports, Everyday Problems Test (EPT), verbal ability, inductive reasoning, and education.

		Age Group								
		Middle-aged			Young-old			Old-old		
Variable		*M*	*SD*	range	*M*	*SD*	range	*M*	*SD*	range
Age		54.61	2.69	50–59	66.73	4.77	60–74	80.96	5.23	75–92
IADL		21.55	3.24	12–24	22.23	2.35	15–24	21.13	2.31	14–24
EPT		17.68	5.07	4–25	15.43	5.28	7–25	13.08	5.12	1–23
Verbal ability		14.61	5.01	5–26	16.20	4.53	7–26	13.33	4.97	5–23
Inductive reasoning		10.76	3.81	3–20	8.58	4.21	3–18	6.58	3.28	3–15
Education		13.71	2.72	7–18	13.02	2.97	4–22	11.40	3.49	6–19
EPT domains										
	Telephone	2.58	0.83	1–4	2.56	0.98	1–4	2.35	0.93	0–4
	Shopping	2.63	1.02	0–4	2.35	0.98	0–4	1.95	1.19	0–4
	Meal Preparation	2.39	1.00	0–4	2.20	1.14	0–4	1.75	0.97	0–3
	Housekeeping	2.61	1.05	0–4	2.30	1.14	0–4	1.85	0.99	0–3
	Transportation	2.39	1.00	0–4	1.90	1.13	0–4	1.45	1.19	0–4
	Medication	2.47	1.06	0–4	2.13	1.14	0–4	1.65	0.99	0–4
	Finances	2.61	1.22	0–4	1.98	1.00	0–4	1.90	1.21	0–4
IADL domains										
	Telephone	2.84	0.44	1–3	2.98	0.16	2–3	3.00	0.00	3–3
	Shopping	2.79	0.53	1–3	2.85	0.36	2–3	2.65	0.59	1–3
	Meal Preparation	2.74	0.50	1–3	2.68	0.52	1–3	2.65	0.49	2–3
	Housekeeping	2.47	0.65	1–3	2.68	0.57	1–3	2.50	0.61	1–3
	Transportation	2.82	0.39	2–3	2.75	0.49	1–3	2.45	0.69	1–3
	Medication	2.84	0.49	1–3	2.95	0.32	1–3	2.85	0.49	1–3
	Finances	2.71	0.61	1–3	2.83	0.50	1–3	2.70	0.73	1–3

Although the sample of community-dwelling middle-aged and older adults reported high levels of IADL abilities, there was also variability within this measure. The majority of the sample (61.76%; *n* = 63) reported that they required some assistance performing the IADL tasks. Participants requiring some IADL assistance ranged in age from 50 to 92. The average age of participants requiring some IADL assistance (*M* = 66.92; *SD* = 11.63) was similar to the total sample (*M* = 65.56; *SD* = 10.94). In addition, the age of participants requiring some IADL assistance was normally distributed.

Since participants were not screened for dementia, the data was examined to determine whether evidence exists that individuals in the sample possibly demonstrated substantial cognitive impairment. The data was also examined to assess whether participants scoring abnormally low on the various cognitive assessments also reported needing some IADL assistance. This would indicate whether participants possessed the self-awareness and understanding to provide valid self-reports of IADL ability.

To assess whether evidence suggested that some participants had cognitive impairment, individuals scoring more than 1.5 *SD* below the mean for the EPT, letter series, and verbal meanings test were examined. For the EPT, 5 participants scored more than 1.5 *SD* below the mean (aged 50 to 75), with total scores ranging from 1 to 7. No participants scored more than 1.5 *SD* below the mean for the letter series test. For the verbal meanings test, 7 participants (ages 54 to 92) scored more than 1.5 *SD* below the mean, with total scores ranging from 5 to 7. One participant (age 58) scored more than 1.5 *SD* below the mean for both the EPT (scoring 4) and the verbal meanings test (scoring 6). This participant also reported a need for IADL assistance related to transportation.

To assess the extent that those individuals scoring low on the EPT and the verbal meanings test were cognitively aware of their abilities, further descriptive analyses examined their IADL scores compared to the remaining sample that did not fall into these low scoring groups. While 58.89% of the remaining sample (*n* = 90) reported requiring some IADL assistance, 83.33% (*n* = 12) of those with a low cognitive score reported some need for assistance related to IADL performance. This data therefore demonstrates that no participants scored more than 1.5 *SD* below the mean for the letter series test, only 1 participant scored more than 1.5 *SD* below the mean on multiple tests, and those with low scores were more likely to report a need for IADL assistance than the rest of the sample.

An examination of gender differences for all of the variables included in the current study revealed no significant sex differences based on EPT performance, the verbal meanings test, the letter series test, or age (*p* > 0.05). There were significant sex differences in self-reported IADL ability, with women (*M* = 22.75; *SD* = 2.01) reporting significantly higher IADL ability than men (*M* = 20.69; *SD* = 2.95) *t*(100) = −4.12; *p* < 0.001.

### 3.2. The Relation between EPT and IADL Scores

To better understand the relation between the measures utilized in the current study and primarily to assess the relation between EPT and IADL scores, Spearman correlations were conducted due to the non-normal distribution in the IADL reports. The results indicated there was a positive significant correlation between the two variables (*r_s_* = 0.20; *p* < 0.05; See Table 2) indicating that higher EPT performance was associated with higher reported functional ability in completing the IADL tasks. The correlations also revealed that EPT scores were positively related to inductive reasoning (*p* < 0.001) and negatively related to age (*p* < 0.001). Verbal ability was also positively related to inductive reasoning (*p* < 0.05). Inductive reasoning was negatively related to age (*p* < 0.001).

An additional set of Spearman correlations were conducted examining the relation between the domain specific EPT scores to the reported IADL ability in the domains of telephone, shopping, meal preparation, housekeeping, and transportation. As can be seen in Table 3, there were significant correlations between EPT and corresponding IADL domains for shopping, meal preparation, housekeeping, and transportation. These significant correlations were larger (*r_s_* = 0.47, 0.34, 0.32, and 0.26) than the significant relation for the overall EPT and IADL scores (*r_s_* = 0.20). There was a trend (*p* = 0.09) when comparing the finance domain for the two measures. The correlations for telephone and medication were not significant.

**Table 2:** Correlations of inductive reasoning, verbal ability, everyday problem solving, instrumental activities of daily living and age.

Variables	2	3	4	5
1. Instrumental Activities of Daily Living	0.20*	0.17	0.08	−0.12
2. Everyday Problem Solving		0.18	0.61***	−0.38***
3. Verbal Ability			0.23*	−0.03
4. Inductive Reasoning				−0.49***
5. Age				

* *p* < 0.05; *** *p* < 0.001.

In addition, some domains of the EPT and IADL measures were related to different domains. Specifically, EPT transportation scores were significantly related to all IADL domains except for shopping and finances. IADL transportation scores were significantly related to all EPT domains except for medication and finances. Aside from these transportation-related correlations, there were only 3 other significant correlations in non-matched domains (EPT housekeeping with IADL shopping, EPT medication with IADL telephone, and EPT medication with IADL finances).

**Table 3:** Correlations of domain specific EPT scores and the corresponding domains of self-reported IADL ability.

	Corresponding IADL scores						
EPT Domains	1	2	3	4	5	6	7
1. Telephone	0.06	0.16	−0.01	0.03	0.26**	−0.05	0.02
2. Shopping	−0.03	0.47***	0.01	0.07	0.45***	0.08	0.13
3. Meal Preparation	0.00	0.18	0.34***	0.19	0.29**	0.13	0.14
4. Housekeeping	−0.01	0.25*	0.09	0.32***	0.37***	0.07	0.08
5. Transportation	−0.22*	0.14	−0.28**	−0.36***	0.26**	−0.23*	−0.14
6. Medication	−0.30**	−0.09	−0.15	−0.05	0.02	0.07	−0.25*
7. Finances	−0.11	0.07	−0.14	−0.19	0.17	−0.18	0.17

* *p* < 0.05; ** *p* < 0.01.; *** *p* < 0.001.

### 3.3. Regression Analyses Predicting Functional Ability

Additional analyses were conducted for the five EPT and IADL domains that demonstrated significant correlations or trends (*i.e*., shopping, meal preparation, housekeeping, transportation, and finances). The purpose of these additional analyses were to examine the extent that specific EPT scores within these domains could predict reported IADL ability in the corresponding domain after accounting for other demographic and cognitive factors (age, sex, verbal ability, inductive reasoning, and total EPT performance). A series of hierarchical regression analyses was conducted. Also, due to the non-normal distribution of IADL data, the regressions were also conducted using various transformations to improve the non-normal data distribution (*i.e*., square root, log, and inverse). The transformed variables did not alter the extent that individual predictors, steps within the model, the total models, or Δ *R^2^* were significant. The data being reported therefore utilizes the non-transformed data with additional notes describing the similarity of each of the regressions to the same analysis using the 3 transformed variables. 

#### 3.3.1. Shopping

The first step of the hierarchical regression predicting self-reports of shopping ability did not reveal any significant relationships between the reported ability and age, sex, verbal ability, inductive reasoning or EPT total (*p* > 0.05). The addition of the shopping EPT score to the model resulted in a significant predictive ability of the model, explaining 26% of the variance in reported shopping ability (*p <* 0.001). The addition of shopping EPT score into the model also resulted in 19% more variance explained compared to the first step in the regression (*p* < 0.001). EPT total score was also a significant individual predictor in the full model (*p* < 0.05; See Table 4). 

**Table 4:** Summary of hierarchical regression analysis predicting reported shopping ability.

Predictors		β	Standardized β	*R^2^*	Δ *R^2^*
Step 1				0.06	
	Age	−0.01	−0.10		
	Sex	−0.06	−0.06		
	Verbal Ability	0.01	0.09		
	Inductive Reasoning	−0.00	−0.02		
	EPT Total	0.01	0.16		
Step 2				0.26***	0.19***
	Age	−0.00	−0.09		
	Sex	0.01	0.01		
	Verbal Ability	0.01	0.06		
	Inductive Reasoning	0.00	−0.00		
	EPT Total	−0.03*	−0.30*		
	EPT Shopping	0.30***	0.65***		

Note: Additional analyses utilizing log, inverse, and square root transformations produced consistent results that did not differ regarding the significance of any individual predictors, model steps, or Δ *R^2^*. The range of Δ *R^2^* using the transformed data was 0.20 to 0.22. * *p* < 0.05; *** *p* < 0.001.

#### 3.3.2. Meal Preparation

Step 1 explained a significant amount of variance (21%; *p* < 0.001) in self-reports of ability related to preparing meals. Within this step, sex was the only significant individual predictor (*p* < 0.001). The coding for sex (men = 1; women = 2) and the positive relation indicated that being female was associated with higher levels of meal preparation ability. In the full model, including the EPT meal preparation score significantly contributed to the predictability of the model by adding an additional 15% of explained variance (*p* < 0.001). Within the full model, sex (*p* < 0.01), total EPT score (*p* < 0.01), and EPT meal preparation (*p* < 0.001) scores were significant predictors of reported meal preparation ability (See Table 5).

**Table 5:** Summary of hierarchical regression analysis predicting reported meal preparation ability.

Predictors		β	Standardized β	*R^2^*	Δ *R^2^*
Step 1				0.21***	
	Age	0.00	−0.01		
	Sex	0.47***	0.47***		
	Verbal Ability	−0.01	−0.11		
	Inductive Reasoning	0.02	0.13		
	EPT Total	0.00	0.00		
Step 2				0.36***	0.15***
	Age	0.00	−0.00		
	Sex	0.32**	0.33**		
	Verbal Ability	−0.02	−0.15		
	Inductive Reasoning	0.02	0.15		
	EPT Total	−0.04**	−0.47**		
	EPT Meal Preparation	0.29***	0.62***		

Note: Additional analyses utilizing log, inverse, and square root transformations produced consistent results that did not differ regarding the significance of any individual predictors, model steps, or Δ *R^2^*. The range of Δ *R^2^* using the transformed data was 0.16 to 0.17. ** *p* < 0.01; *** *p* < 0.001.

#### 3.3.3. Housekeeping

Reported housekeeping ability was also significantly related to sex (*p* < 0.001), indicating that being a women was associated with higher reported levels of housekeeping ability. This significant correlation resulted in a significant first step of the model (*p* < 0.01), explaining 18% of the variance in reported housekeeping ability. The second step added significantly to the model (*p* < 0.001), explaining an additional 19% of the variance in reported housekeeping ability. Sex (*p* < 0.001), EPT total (*p* < 0.01), and EPT housekeeping score (*p* < 0.001), were significant individual predictors within the full model (See Table 6).

**Table 6:** Summary of hierarchical regression analysis predicting reported housekeeping ability.

Predictors		β	Standardized β	*R^2^*	Δ *R^2^*
Step 1				0.18**	
	Age	0.00	0.03		
	Sex	0.47***	0.39***		
	Verbal Ability	0.01	0.08		
	Inductive Reasoning	−0.02	−0.13		
	EPT Total	0.01	0.11		
Step 2				0.37***	0.19***
	Age	0.00	0.05		
	Sex	0.40***	0.34***		
	Verbal Ability	0.01	0.05		
	Inductive Reasoning	−0.01	−0.09		
	EPT Total	−0.05**	−0.41**		
	EPT Housekeeping	0.37***	0.67***		

Note: Additional analyses utilizing log, inverse, and square root transformations produced consistent results that did not differ regarding the significance of any individual predictors, model steps, or Δ *R^2^*. The range of Δ *R^2^* using the transformed data was 0.19 to 0.20. ** *p* < 0.01; *** *p* < 0.001.

#### 3.3.4. Transportation

EPT transportation scores did not add significantly to the model predicting reported transportation ability (*p* > 0.05), adding less than 1% to the explained variance. The first step of the model, however, did predict reported transportation ability scores, explaining 22% of the variance, with sex as a significant individual predictor (*p* < 0.05) of self-reported transportation ability. Being male was associated with higher levels of reported transportation ability (see Table 7). 

**Table 7:** Summary of hierarchical regression analysis predicting reported transportation ability.

Predictors		β	Standardized β	*R^2^*	Δ *R^2^*
Step 1				0.22***	
	Age	−0.01	−0.19		
	Sex	−0.23*	−0.21*		
	Verbal Ability	0.01	0.12		
	Inductive Reasoning	0.01	0.05		
	EPT Total	0.02	0.20		
Step 2				0.23***	0.00
	Age	−0.01	−0.19		
	Sex	−0.24*	−0.22*		
	Verbal Ability	0.01	0.12		
	Inductive Reasoning	0.01	0.05		
	EPT Total	0.02	0.24		
	EPT Transportation	−0.03	−0.06		

Note: Additional analyses utilizing log, inverse, and square root transformations produced consistent results that did not differ regarding the significance of any individual predictors, model steps, or Δ *R^2^*. The Δ *R^2^* using the transformed data was 0.00 for all three transformations. * *p* < 0.05; *** *p* < 0.001.

#### 3.3.5. Finances

Step 1 (explaining 5% of the variance) and the full model (explaining 9% of the variance) did not significantly predict reported ability related to finances (*p* > 0.05). EPT finance score, however, was a significant individual predictor (*p* < 0.05), resulting in a significant Δ *R^2^*, explaining an additional 4% of the variance (See Table 8). 

**Table 8:** Summary of hierarchical regression analysis predicting reported finances ability.

Predictors		β	Standardized β	*R^2^*	Δ *R^2^*
Step 1				0.05	
	Age	0.00	0.01		
	Sex	0.15	0.12		
	Verbal Ability	0.00	−0.00		
	Inductive Reasoning	0.03	0.22		
	EPT Total	−0.01	−0.06		
Step 2				0.09	0.04*
	Age	0.00	−0.01		
	Sex	0.14	0.11		
	Verbal Ability	0.00	0.01		
	Inductive Reasoning	0.02	0.17		
	EPT Total	−0.03	−0.24		
	EPT Finances	0.15*	0.30*		

Note: Additional analyses utilizing log, inverse, and square root transformations produced consistent results that did not differ regarding the significance of any individual predictors, model steps, or Δ *R^2^*. The Δ *R^2^* using the transformed data was 0.04 for all three transformations. * *p* < 0.05.

## 4. Conclusions

Although the current study did find a significant correlation between everyday problem-solving and functional ability, findings also suggest a potential relation between these variables within domains. On a bivariate level, the correlations tended to be stronger when implementing a domain-specific approach for 4 of the 7 domains (shopping, meal preparation, housekeeping, and transportation). In addition, the regression analyses revealed that the domain specific EPT performance explained a significant amount of variance in reported IADL ability in corresponding domains (even after accounting for age, sex, verbal ability, inductive reasoning, and total EPT score) for shopping, meal preparation, housekeeping, and finances. This domain-specific focus suggests that middle-aged and older adults may vary regarding their cognitive and physical abilities related to specific IADL tasks. 

### 4.1. Research Implications

Previous research has provided evidence suggesting that different types of knowledge and skills may be required for performing different IADL tasks. Although IADLs require a combination of both physical and cognitive abilities, Ng, Niti, Chiam, and Kua [20] suggested that some IADLs tended to depend more on physical attributes while other IADLs are more dependent on cognitive attributes. 

The current study also suggests a potential benefit from focusing on more specific IADL tasks instead of overall IADL scores. In the current study, this domain-specific approach tended to demonstrate a relation between EPT and IADL tasks even after accounting for other aspects of cognitive ability, including total EPT scores. This suggests that experience-based knowledge relevant to specific domains of IADL performance could assist individuals’ ability to carry out these tasks. 

Surprisingly, the two domains that did not demonstrate a significant relation between corresponding domains of functional ability and everyday problem-solving performance were the telephone use and the health / medication domains. This was unexpected considering Ng, Niti, Chiam, and Kua’s [20] research identified three IADLs that were more dependent on cognitive abilities. These cognitive IADLs included telephone use, finances, and medication use. Two of these cognitive IADL tasks (telephone use and medication use) were the only domains that did not relate to everyday problem-solving performance within the same domain. It would be expected that the cognitive focus of these tasks and the limited dependence on physical abilities would result in a stronger association with the corresponding everyday problem-solving domains. 

A closer look at the EPT items used for these domains could help explain this discrepancy. First, the two stimuli relating to telephone use included interpreting a telephone bill and estimating the cost of calls based on a telephone rate schedule that varied with the time of day. Although these tasks are telephone related, calculating the costs of a specific calls and interpreting an itemized bill are not skills that are typically required for successful telephone use. It is therefore likely that reports of functional ability related to telephone use were referring to different types of telephone skills (such as finding numbers, making calls, and managing messages), while the EPT items were mostly concerned with telephone billing issues.

Regarding medication use, one of the EPT items does appear closely related to this IADL concept. Participants were presented with a label for an over-the-counter cough medication and were asked specific questions about the proper use of this medication. The other item in the EPT health/medication domain is less related to medication use as participants are asked questions related to a medical history form, similar to those completed prior to a visit with a physician. Still, exploratory analyses revealed that using only these medication-based items from the EPT did not result in a significant correlation between the EPT and IADL domains (*r_s_* = 0.01; *p* > 0.05). It is possible that older adults may self-report their medication use skills based more on their experiences with managing prescription drug schedules. These schedules may include more explicit instructions and less ambiguity allowing older adults to plan a regimented medication schedule throughout the week. The over-the-counter label used in the EPT, however, provided information describing situational characteristics related to when the medication should be avoided.

Another unusual finding in this study was the lack of a significant relation between reported functional ability and age. Previous research has demonstrated that advanced age is associated with decreased ability performing IADL tasks and that this age related change is revealed in self-report measures [27]. The lack of a relation between IADLs and age could be due to unique sample characteristics. Data was mostly collected in rural areas where many participants currently or previously held jobs requiring manual labor (such as coal miners). During informal discussion with participants following participation in the study, several participants discussed physical disability issues caused by job-related injuries. Many also mentioned that they were unable to work due to injury-related physical disabilities. Physical disability status was not an a priori focus of the current study and is therefore only anecdotally discussed. If, however, the sample consisted of a disproportionate amount of participants with physical limitations (particularly in the younger age groups), decreasing their functional ability, the relation between age and functional ability could have been affected by this confounding variable. Also, an examination of the correlations between age and performance on cognitive tasks (inductive reasoning, EPT, and verbal ability; see Table 2), suggests that the anticipated relations were found. The sample therefore appears to be normative regarding the relation between cognitive performance and age. These significant relations suggest that the lack of relation between age and self-reported functional ability may possibly be due to a potential confound (such as physical disability status) affecting the physical demands of IADL tasks.

### 4.2. Applied Implications

Lawton and Brody’s self-reported IADL measure [21] is an efficient assessment of functional ability that can be used in a variety of clinical settings [33]. Previous research has suggested that this assessment could benefit from acknowledging different physical and cognitive demands required to complete different IADL tasks [20]. The current study also provides evidence supporting a more domain-specific approach to IADL assessments. When using this measure in applied settings, it may therefore be beneficial to focus on scores within each domain opposed to looking at a patient’s overall score as a more global indicator of functional ability. For example, an individual may lack ability in one domain while being very independent in all other domains. A total score would indicate little need for intervention while the domain-specific score would indicate a clear need for assistance in one specific area. Also, as noted by Graff [33], some low scores could be due to situational variables such as the loss of a spouse that previously took responsibility for a specific activity. Older adults may also provide overly positive reports if they fear losing their independence. It would therefore be beneficial to conduct further assessments to obtain additional information (possibly from third parties) regarding the extent of and potential reasons for low functional ability within specific domains.

### 4.3. Limitations

There were several limitations within the current study. First, the use of self-report to assess functional ability could be problematic. As previously mentioned, this measure could result in participants providing an overly-positive report of their functional abilities. Although the Lawton and Brody [21] IADL measure is one of the most commonly used assessments of functional ability and has been found to reliably predict a variety of outcomes, this inherent limitation of self-report measures persists. Another limitation is the cross-sectional nature of the current study. It is possible that any age related findings could have also been influence by cohort effects in addition to participant age. Also, this sample consisted of a relatively high functioning group of community dwelling adults representing a large age range (50–92). Although other research [27] has utilized similar large age ranges (50 and older) to examine relations between cognitive ability, everyday problem solving, and IADL status, the findings may not generalize to an older and more disabled (both cognitively and physically) population. Although the majority of the sample reported some IADL needs, given the relatively young and high functioning characteristics of this sample, a more sensitive measure of IADL ability may have been useful in detecting more subtle declines in this sample. Future research is therefore needed to assess the extent that these findings generalize to older and lower functioning samples and the extent that these findings would generalize to more sensitive IADL measures. 

Additionally, participants were not screened for general mental status or cognitive impairment. Data presented in the descriptive section of the results suggests that individuals tended to not score exceptionally low on multiple cognitive measures. Also, low scores tended to be associated with reports of need for IADL assistance, suggesting that those with lower cognitive abilities were still aware of their inability performing these tasks. Still, it cannot be assumed that no participants in the current study suffered from cognitive impairment. Another limitation was the specific nature of the regression analyses that were conducted. The purpose of theses analyses was to demonstrate the extent that domain specific IADLs were predicted by the corresponding EPT score after accounting for other variables including total EPT score. As indicated by the correlations presented in Table 3, there were some significant relationships among non-matched EPT and IADL domains. The regression analyses do not exclude the possibility of non-corresponding EPT domains accounting for variance in domain specific IADL scores. Finally, the lack of a relation between age and functional ability suggests that this sample could have some unique characteristics, potentially limiting the generalizability of these findings.

### 4.4. Future Directions

The findings from the current study suggest several potential areas of future research. More studies should investigate the potential implications of a domain-specific focus on both functional ability and everyday problem solving. Specifically, the extent that these domain-specific measures predict future decline within those and other domains should be investigated. Also, more information is needed regarding the extent that prior experiences and basic cognitive abilities interact to predict domain-specific functional ability. Observationally-based assessments of functional ability could also be examined to determine the extent that domain-specific everyday problem-solving and self-reported functional ability predict observed ability in the corresponding and other domains.

Future studies examining the relation between EPT and IADL scores should also account for the order that these measures are administered. In the current study, the EPT test was administered before the reports of IADL ability. This order was selected based on previous research that suggests older adults may perform worse on cognitive tasks when they are given information that could suggest potential age-related stereotypes or age-related declines [35]. The effects of these types of suggestions may have a greater effect with increasing age. The IADL questionnaire could therefore prime older adults to consider age-related declines or negative age-related stereotypes that might result in poorer performance on the EPT. It is possible, however, that difficulties experienced during the completion of the EPT could affect subsequent reports of IADL ability. In the current study, this cannot be ruled out. It is important to note, however, that reported IADL ability in this study was high and the anticipated relation between age and IADL reports was not found. EPT scores, however, varied considerably and were correlated with age. Therefore, if EPT performance were to affect the IADL reports, this would increase the likelihood of finding the anticipated link between IADL ability and age. Still, future research should consider counterbalancing these measures to assess whether one measure may influence the scores of the other.

The findings related to sex and reported IADL ability (specifically related to meal preparation and housekeeping) could also be clarified in future studies. The tendency for men to report lower ability in these domains could be due to gender norms within older cohorts. The reported need of assistance in these domains could be less related to a declining ability and more related to a lack of familiarity, knowledge, and experience related to these specific tasks. Future research should attempt to better clarify the extent that these reports are due to declining ability and a lack of experience. In either case, the tendency for men to report a lower ability in these tasks along with a greater need of assistance is important. Intervention attempts, however, would vary in these different scenarios. Instances of low reported ability that are more linked to declining functional ability would require more efforts related to potential compensatory strategies. Instances of low reported ability that are due to a lack of experience and knowledge could be changed with more of an educational approach focusing on familiarizing individuals with these tasks. 

Another area for future research involves the extent that domain-specific IADL performance is affected by social contexts. Previous studies suggest that performance on the EPT can be improved through interactive processes with social partners such as a spouse [36]. These findings are only relevant to the cognitive components of these IADL domains. Future research should examine the role of social interactions and relationships on both the cognitive and physical components of functional ability in old age. Research should also examine how individuals deal with domain-specific tasks after the loss of a long-term social partner, such as a spouse, that previously assumed responsibility for that specific task. 

### 4.5. Cognition

This study provides further evidence illustrating a need to continue conceptualizing cognitive abilities as distinct skills and processes with different influences and developmental trajectories. While many areas of research focus on general cognitive ability (*g*), research examining cognitive ability within adult development suggests a need to focus more specifically on distinct cognitive abilities (e.g., crystallized, fluid, memory, executive functioning, and processing speed). This study supports the idea of focusing on different specific cognitive abilities and also suggests potential benefits of adopting an even more specific strategy when attempting to link cognitive ability to multifaceted tasks such as IADLs. This is not suggesting that separate, unrelated cognitive abilities are required to perform each individual task that people encounter. Instead, it is likely that culture and experience based (crystallized) knowledge may predispose some individuals to successfully complete certain cognitively demanding tasks while leaving other individuals at a disadvantage. The findings in the current study suggest that domain specific performance on a cognitive task (the EPT) tended to relate to reported ability within the same domain. This could be due to individuals being more experienced in some domains and less experienced in others. This experience could result in the acquisition of domain specific knowledge, information, and strategies that could be used to successfully overcome difficult cognitive components of task performance. This knowledge could also translate to an increased ability to complete these tasks in their day-to-day life. For example, if someone has a great deal of meal preparation experience, they have likely acquired domain-specific information that would help them solve problems related to a challenging task in this area. When presented with a complicated recipe, they are more likely to be familiar with some of the terminology, the general layout of a recipe, the different types of ingredients, and different procedures required for successful completion of the dish. When given a specific question about the recipe (such as in the EPT), this experienced cook would be more likely to be able to find the relevant and correct information. Someone with very little experience in this domain would have a more difficult time sorting through irrelevant information to answer a specific question about a complicated recipe. Essentially, the types of challenges associated with everyday problem-solving tasks are not independent from experience-based knowledge. A general measure of crystallized abilities (such as vocabulary), is less likely to tap into this experienced-based knowledge related to specific domains. A more domain specific approach could therefore assist in determining if an individual has the experience-based knowledge that would predict the successful completion of specific tasks.

The approach used to define cognition is very relevant to the findings and interpretation of the current study. The more specific term, cognitive ability, typically refers to behavioral outcomes related to the completion of challenging tasks. More generally, cognition involves a variety of mental activities and processes related to the acquisition and storage of information. It also includes processes related to utilizing this information such as solving problems and making decisions. Often these processes are inferred based on behavioral outcomes (such as performance on a test). The extent that a variable or construct should be considered cognitive should be determined by the extent that it is intended to provide evidence for these processes. The current study involved several cognitive measures. The tasks related to inductive reasoning and verbal ability are commonly used as indicators of cognitive abilities. Cognitive everyday problem-solving tasks are also related to cognition. Similar to more traditional cognitive measure, these measures assess an individual’s ability to solve challenging problems. These tasks involve familiar domains, however, suggesting that culture and experience-based knowledge may be more influential compared to completing cognitive assessments that use more novel stimuli. 

One component of this study, and whether or not it should be considered part of cognition, is less clear. The self-report method used to assess functional or IADL ability could potentially be considered cognition. The measure is not directly assessing individuals’ ability. Instead, it is assessing participants’ perceived ability. This is an interpretation of their actual ability to perform the given tasks that relies on their utilization of information that they have acquired about their performance on the task. It could therefore be argued that this is a cognitive measure. To define this variable as cognitive, however, would also suggest that any self-report measure across all areas of psychology (and other research disciplines) were cognitive assessments. While this interpretation could be rationally supported, it would broaden the scope of this branch of psychology to an extent that it would lose focus and meaning. Taken to an extreme, this would result in referring to whether or not a participant indicated that they are male or female as a cognitive measure as well. The current study did not refer to the self-reported IADL ability as cognition or a cognitive measure because it was intended to be an indicator of participants’ mental processes linked to an ability to perform a variety of tasks. These tasks consist of both cognitive (e.g., solving task related problems and making task-related decisions) and physical (e.g., having the ability to physically carry-out the task once the cognitive components of the task are complete) demands. The self-report method is considered to be subjective and indirect, but is intended to provide insight related to the task being assessed, not insight to components of processing and interpreting information related to generating a particular self-report response. Other research [20,22] has been consistent with this approach and typically refers to self-reported IADL ability as an indirect measure of actual performance on tasks requiring a combination of physical and cognitive elements.

Although many components of cognition were not included in the current research, this study supports studying cognition as several separate, yet potentially related processes involved with obtaining and utilizing information. An attempt to overly consolidate diverse and multifaceted characteristics of cognition could result in a loss of sensitivity and precision in research along with a decreased ability to predict and understand both cognitive and behavioral correlates and outcomes.
